# Supplementary material for: Seasonal Variation in Cell Wall Composition and Carbohydrate Metabolism in the Seagrass Posidonia oceanica Growing at Different Depths
Source: Plants (Basel). 2023 Sep 1;12(17):3155. doi: 10.3390/plants12173155 (PMC10490095; doi:10.3390/plants12173155)
Supplement: Supplementary file 1 [file plants-12-03155-s001.zip › plants-2299009-supplementary.pptx]

## Slide 1
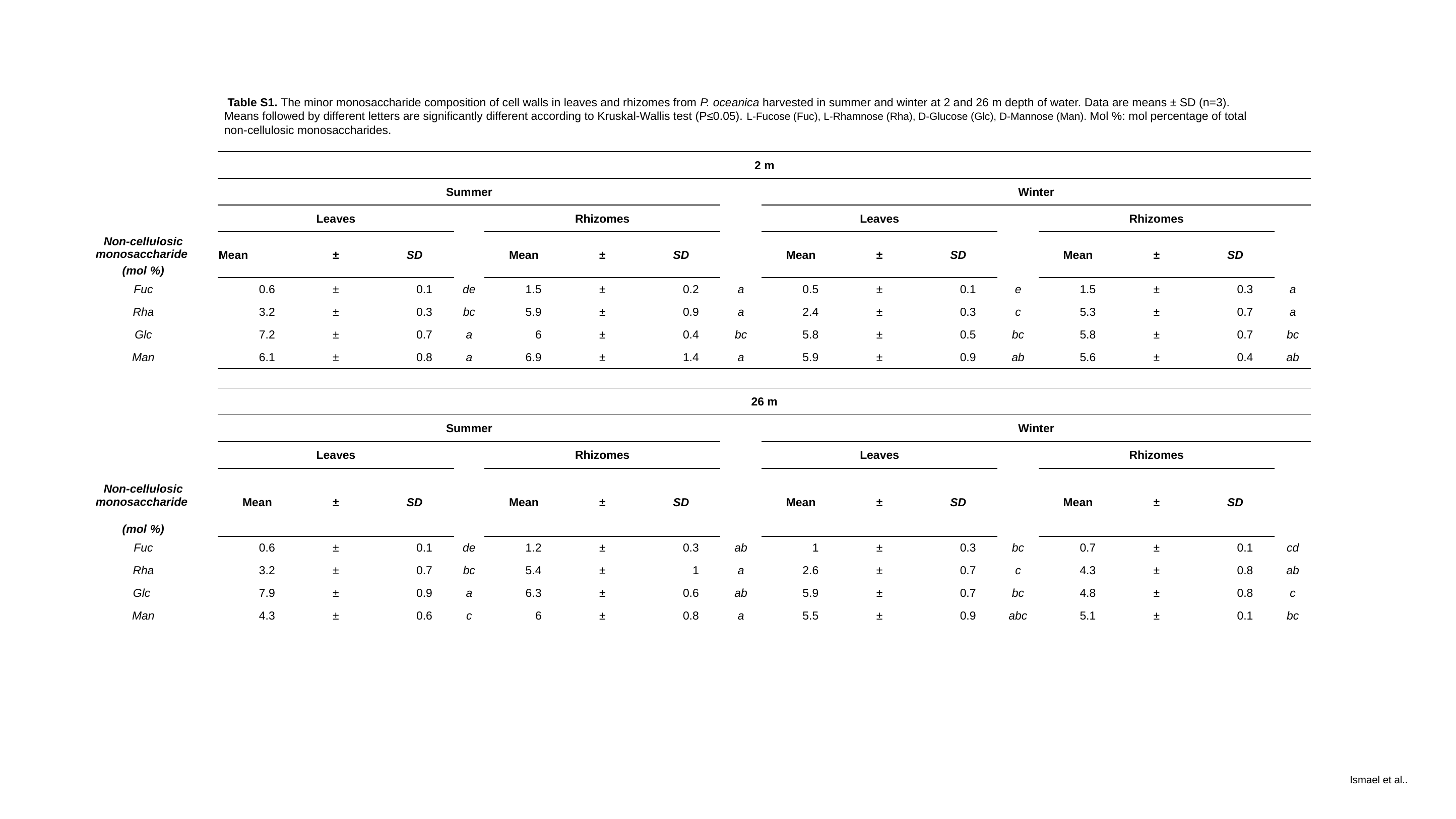

Table S1. The minor monosaccharide composition of cell walls in leaves and rhizomes from P. oceanica harvested in summer and winter at 2 and 26 m depth of water. Data are means ± SD (n=3). Means followed by different letters are significantly different according to Kruskal-Wallis test (P≤0.05). l-Fucose (Fuc), l-Rhamnose (Rha), d-Glucose (Glc), d-Mannose (Man). Mol %: mol percentage of total non-cellulosic monosaccharides.
| | 2 m | | | | | | | | | | | | | | | |
| --- | --- | --- | --- | --- | --- | --- | --- | --- | --- | --- | --- | --- | --- | --- | --- | --- |
| | Summer | | | | | | | | Winter | | | | | | | |
| | Leaves | | | | Rhizomes | | | | Leaves | | | | Rhizomes | | | |
| Non-cellulosic monosaccharide | Mean | ± | SD | | Mean | ± | SD | | Mean | ± | SD | | Mean | ± | SD | |
| (mol %) | | | | | | | | | | | | | | | | |
| Fuc | 0.6 | ± | 0.1 | de | 1.5 | ± | 0.2 | a | 0.5 | ± | 0.1 | e | 1.5 | ± | 0.3 | a |
| Rha | 3.2 | ± | 0.3 | bc | 5.9 | ± | 0.9 | a | 2.4 | ± | 0.3 | c | 5.3 | ± | 0.7 | a |
| Glc | 7.2 | ± | 0.7 | a | 6 | ± | 0.4 | bc | 5.8 | ± | 0.5 | bc | 5.8 | ± | 0.7 | bc |
| Man | 6.1 | ± | 0.8 | a | 6.9 | ± | 1.4 | a | 5.9 | ± | 0.9 | ab | 5.6 | ± | 0.4 | ab |
| | | | | | | | | | | | | | | | | |
| | 26 m | | | | | | | | | | | | | | | |
| | Summer | | | | | | | | Winter | | | | | | | |
| | Leaves | | | | Rhizomes | | | | Leaves | | | | Rhizomes | | | |
| Non-cellulosic monosaccharide | Mean | ± | SD | | Mean | ± | SD | | Mean | ± | SD | | Mean | ± | SD | |
| (mol %) | | | | | | | | | | | | | | | | |
| Fuc | 0.6 | ± | 0.1 | de | 1.2 | ± | 0.3 | ab | 1 | ± | 0.3 | bc | 0.7 | ± | 0.1 | cd |
| Rha | 3.2 | ± | 0.7 | bc | 5.4 | ± | 1 | a | 2.6 | ± | 0.7 | c | 4.3 | ± | 0.8 | ab |
| Glc | 7.9 | ± | 0.9 | a | 6.3 | ± | 0.6 | ab | 5.9 | ± | 0.7 | bc | 4.8 | ± | 0.8 | c |
| Man | 4.3 | ± | 0.6 | c | 6 | ± | 0.8 | a | 5.5 | ± | 0.9 | abc | 5.1 | ± | 0.1 | bc |
Ismael et al..

## Slide 2
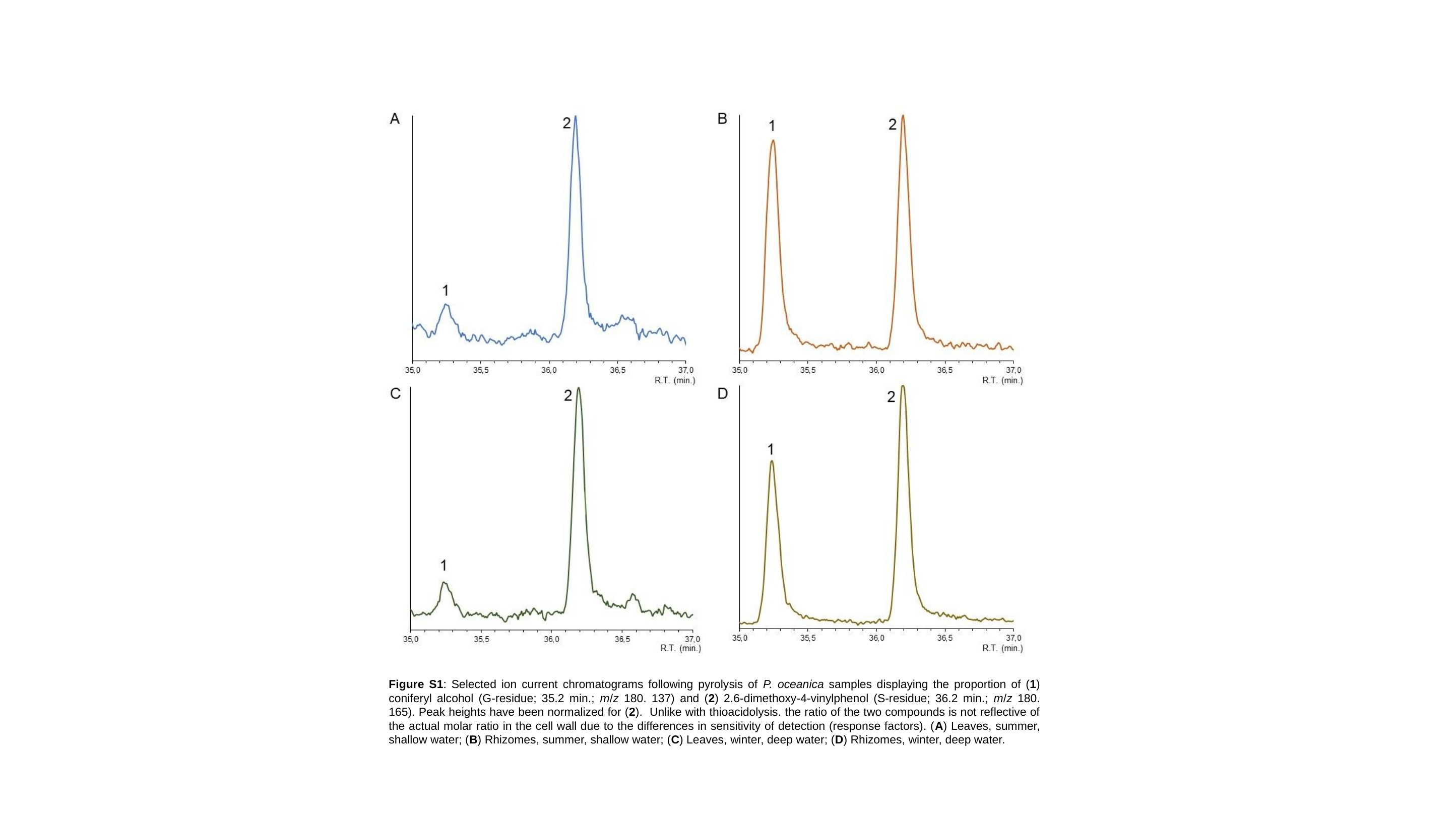

Figure S1: Selected ion current chromatograms following pyrolysis of P. oceanica samples displaying the proportion of (1) coniferyl alcohol (G-residue; 35.2 min.; m/z 180. 137) and (2) 2.6-dimethoxy-4-vinylphenol (S-residue; 36.2 min.; m/z 180. 165). Peak heights have been normalized for (2). Unlike with thioacidolysis. the ratio of the two compounds is not reflective of the actual molar ratio in the cell wall due to the differences in sensitivity of detection (response factors). (A) Leaves, summer, shallow water; (B) Rhizomes, summer, shallow water; (C) Leaves, winter, deep water; (D) Rhizomes, winter, deep water.

## Slide 3
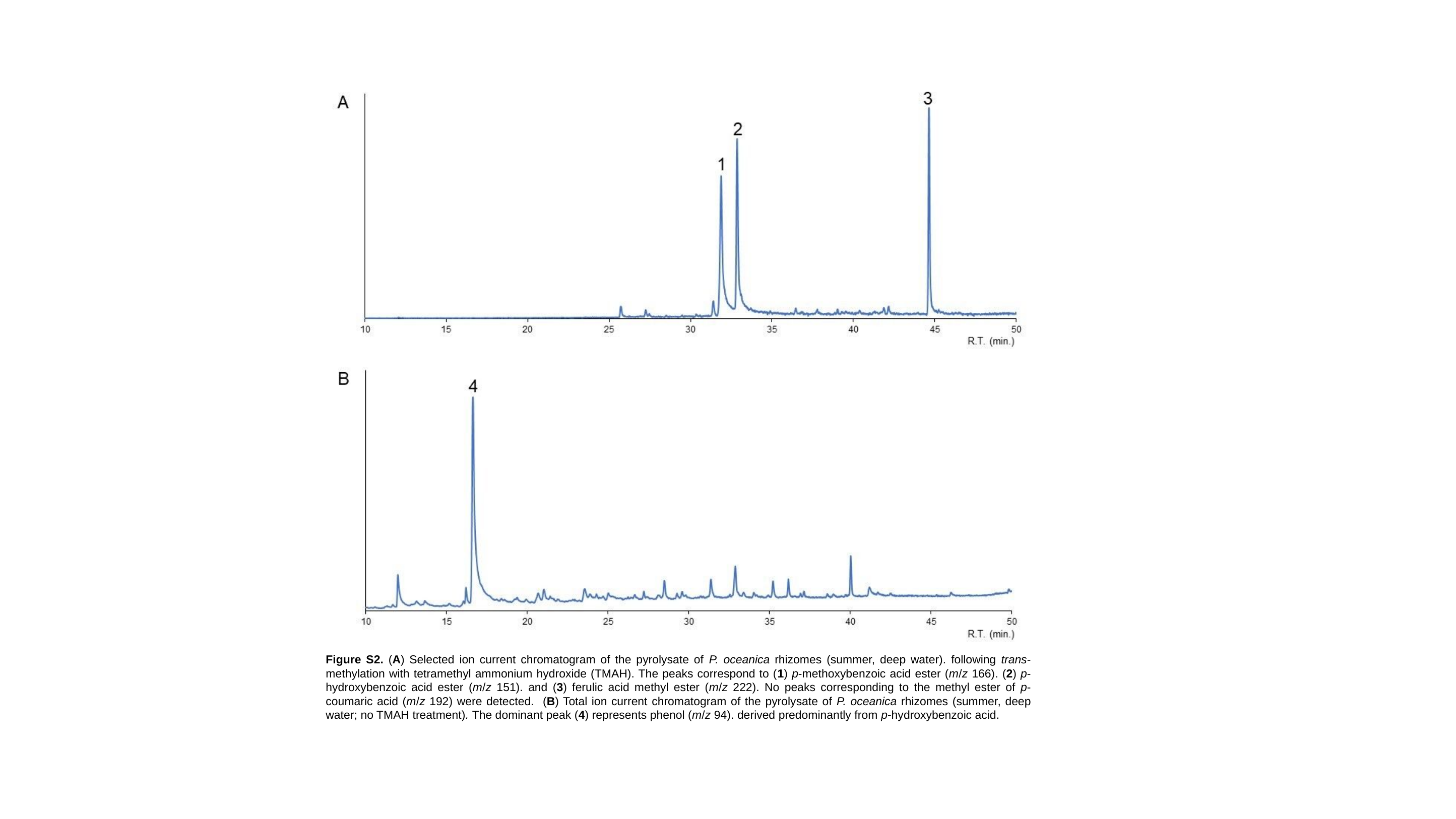

Figure S2. (A) Selected ion current chromatogram of the pyrolysate of P. oceanica rhizomes (summer, deep water). following trans-methylation with tetramethyl ammonium hydroxide (TMAH). The peaks correspond to (1) p-methoxybenzoic acid ester (m/z 166). (2) p-hydroxybenzoic acid ester (m/z 151). and (3) ferulic acid methyl ester (m/z 222). No peaks corresponding to the methyl ester of p-coumaric acid (m/z 192) were detected. (B) Total ion current chromatogram of the pyrolysate of P. oceanica rhizomes (summer, deep water; no TMAH treatment). The dominant peak (4) represents phenol (m/z 94). derived predominantly from p-hydroxybenzoic acid.

## Slide 4
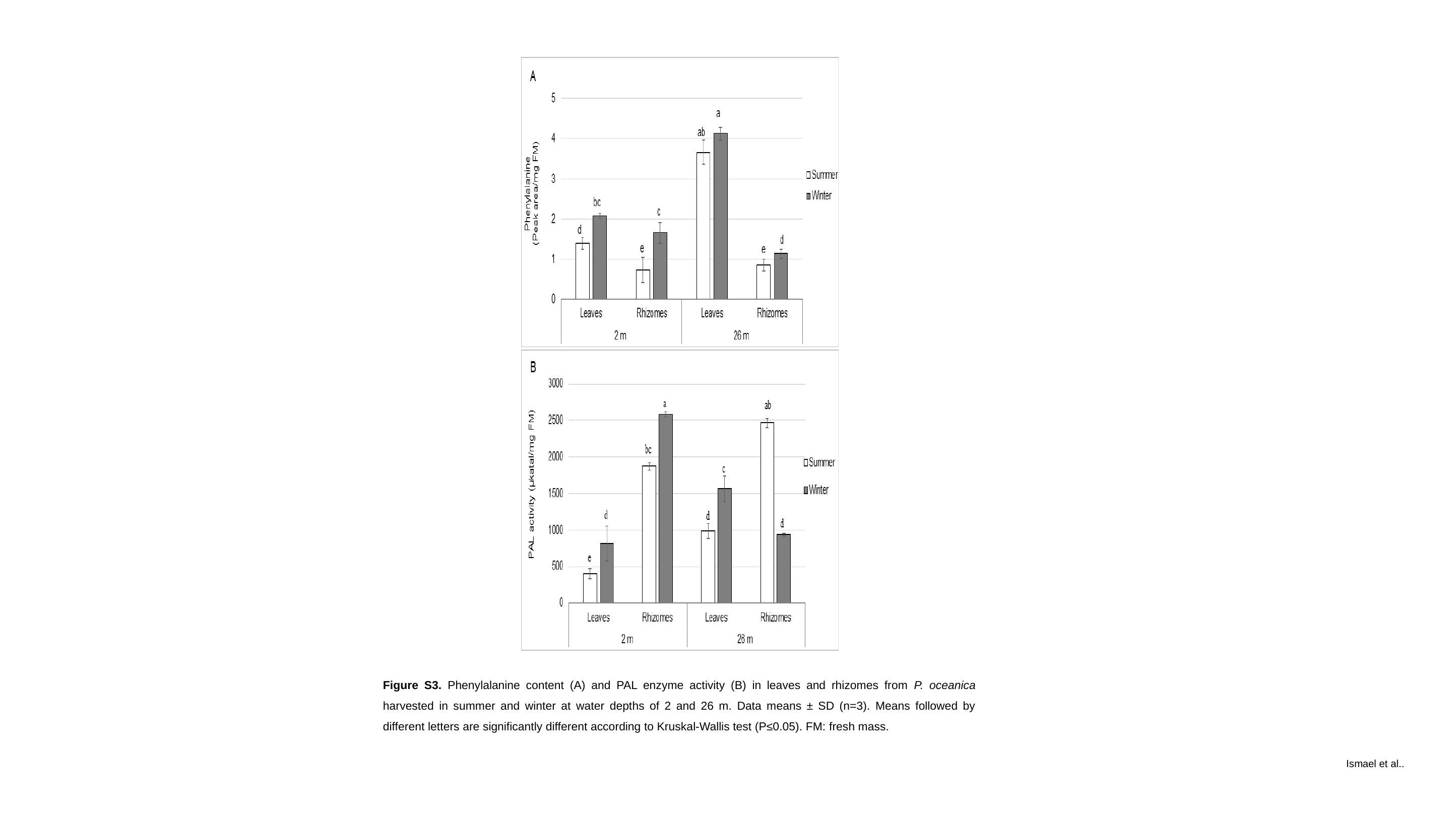

Figure S3. Phenylalanine content (A) and PAL enzyme activity (B) in leaves and rhizomes from P. oceanica harvested in summer and winter at water depths of 2 and 26 m. Data means ± SD (n=3). Means followed by different letters are significantly different according to Kruskal-Wallis test (P≤0.05). FM: fresh mass.
Ismael et al..

## Slide 5
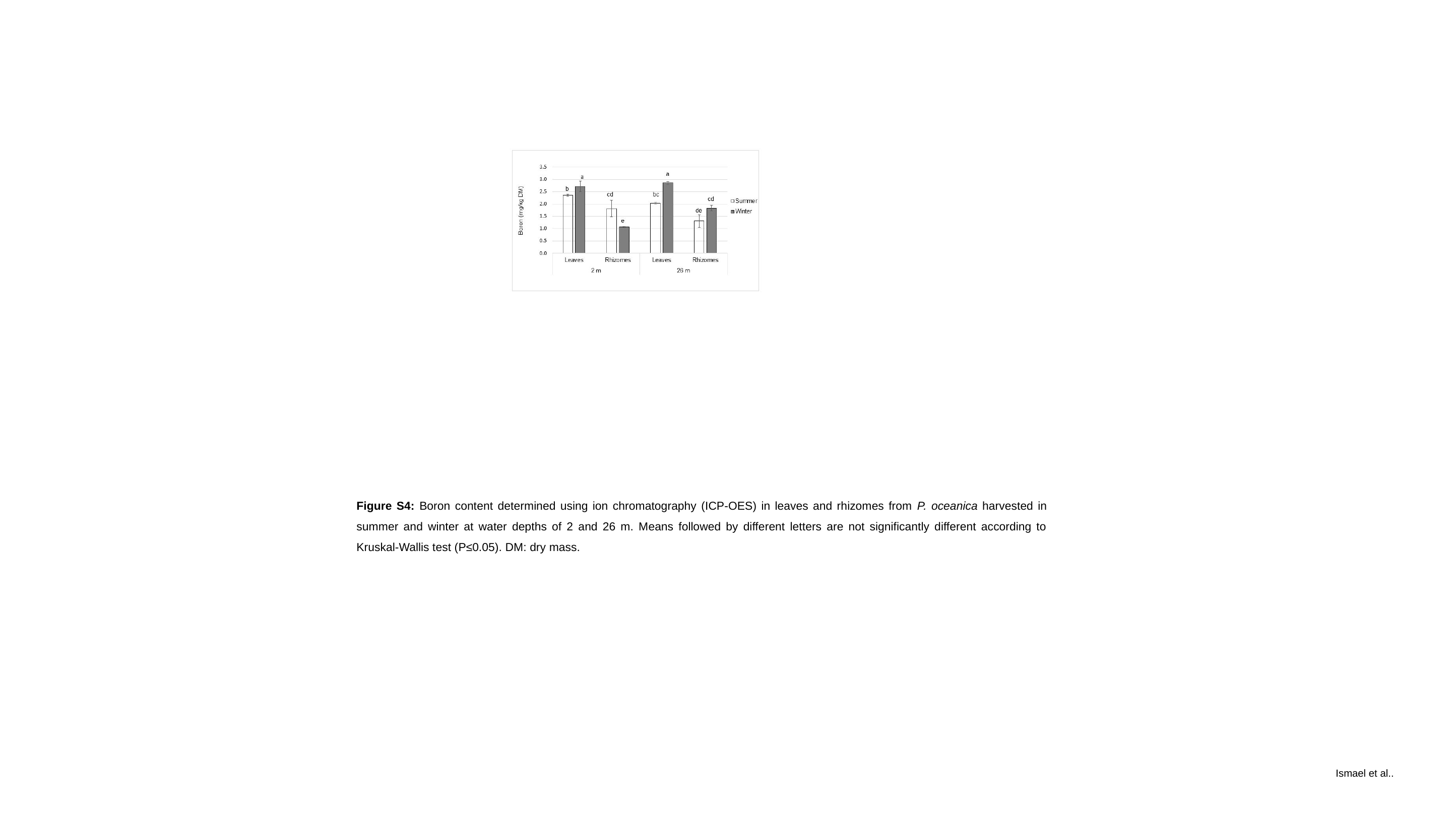

Figure S4: Boron content determined using ion chromatography (ICP-OES) in leaves and rhizomes from P. oceanica harvested in summer and winter at water depths of 2 and 26 m. Means followed by different letters are not significantly different according to Kruskal-Wallis test (P≤0.05). DM: dry mass.
Ismael et al..

## Slide 6
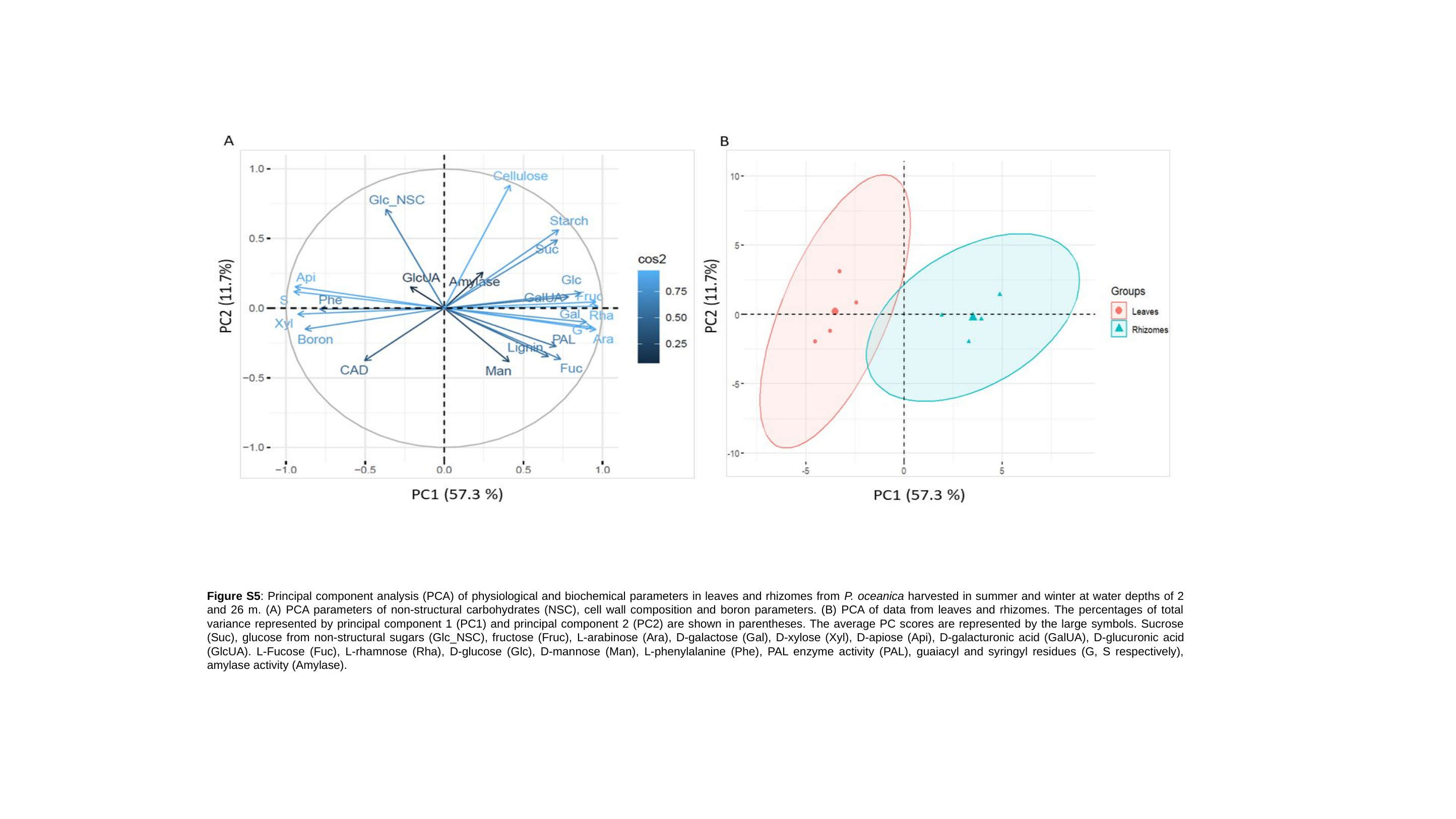

Figure S5: Principal component analysis (PCA) of physiological and biochemical parameters in leaves and rhizomes from P. oceanica harvested in summer and winter at water depths of 2 and 26 m. (A) PCA parameters of non-structural carbohydrates (NSC), cell wall composition and boron parameters. (B) PCA of data from leaves and rhizomes. The percentages of total variance represented by principal component 1 (PC1) and principal component 2 (PC2) are shown in parentheses. The average PC scores are represented by the large symbols. Sucrose (Suc), glucose from non-structural sugars (Glc_NSC), fructose (Fruc), l-arabinose (Ara), d-galactose (Gal), d-xylose (Xyl), d-apiose (Api), d-galacturonic acid (GalUA), d-glucuronic acid (GlcUA). l-Fucose (Fuc), l-rhamnose (Rha), d-glucose (Glc), d-mannose (Man), l-phenylalanine (Phe), PAL enzyme activity (PAL), guaiacyl and syringyl residues (G, S respectively), amylase activity (Amylase).

## Slide 7
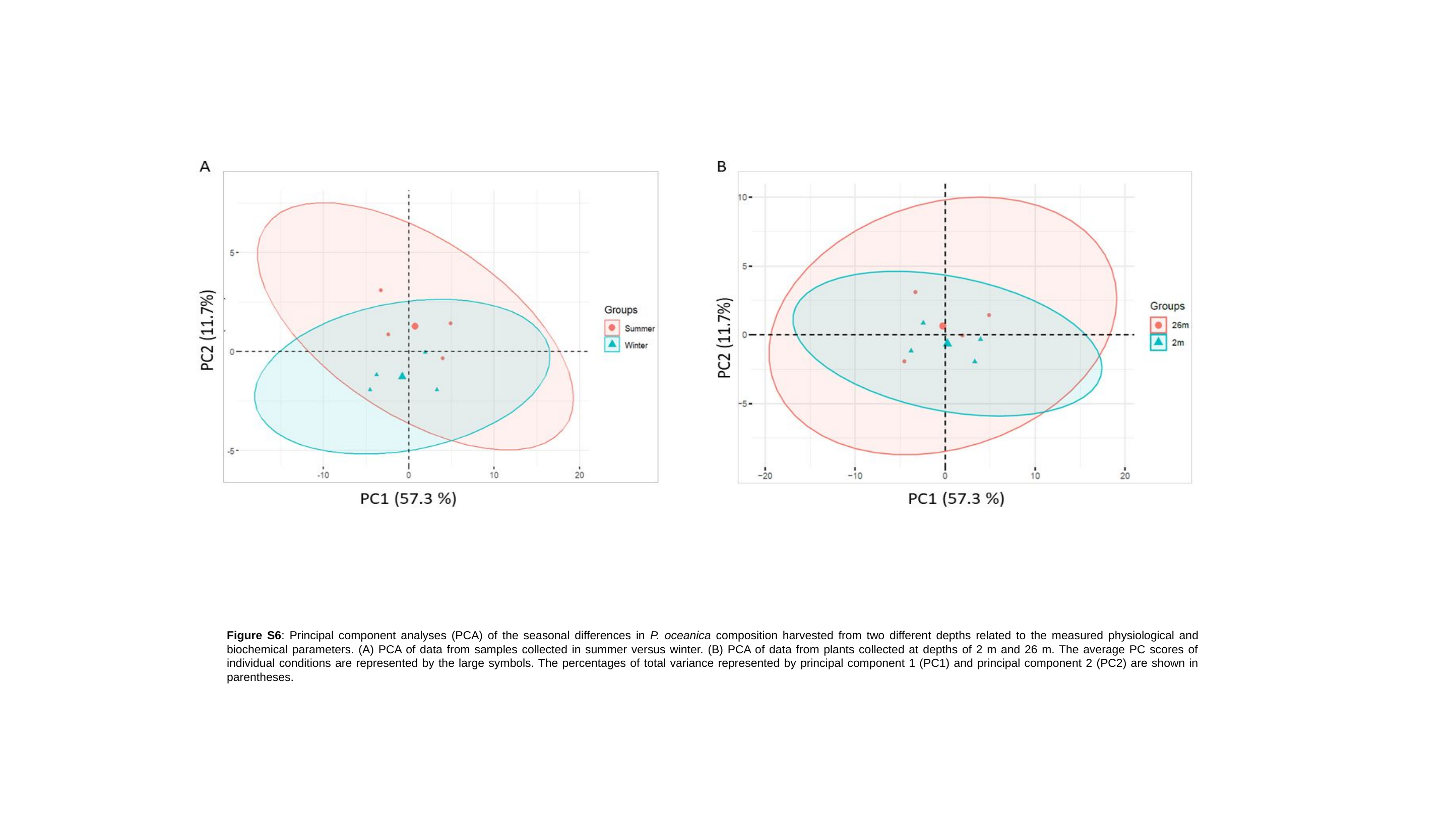

Figure S6: Principal component analyses (PCA) of the seasonal differences in P. oceanica composition harvested from two different depths related to the measured physiological and biochemical parameters. (A) PCA of data from samples collected in summer versus winter. (B) PCA of data from plants collected at depths of 2 m and 26 m. The average PC scores of individual conditions are represented by the large symbols. The percentages of total variance represented by principal component 1 (PC1) and principal component 2 (PC2) are shown in parentheses.

## Slide 8
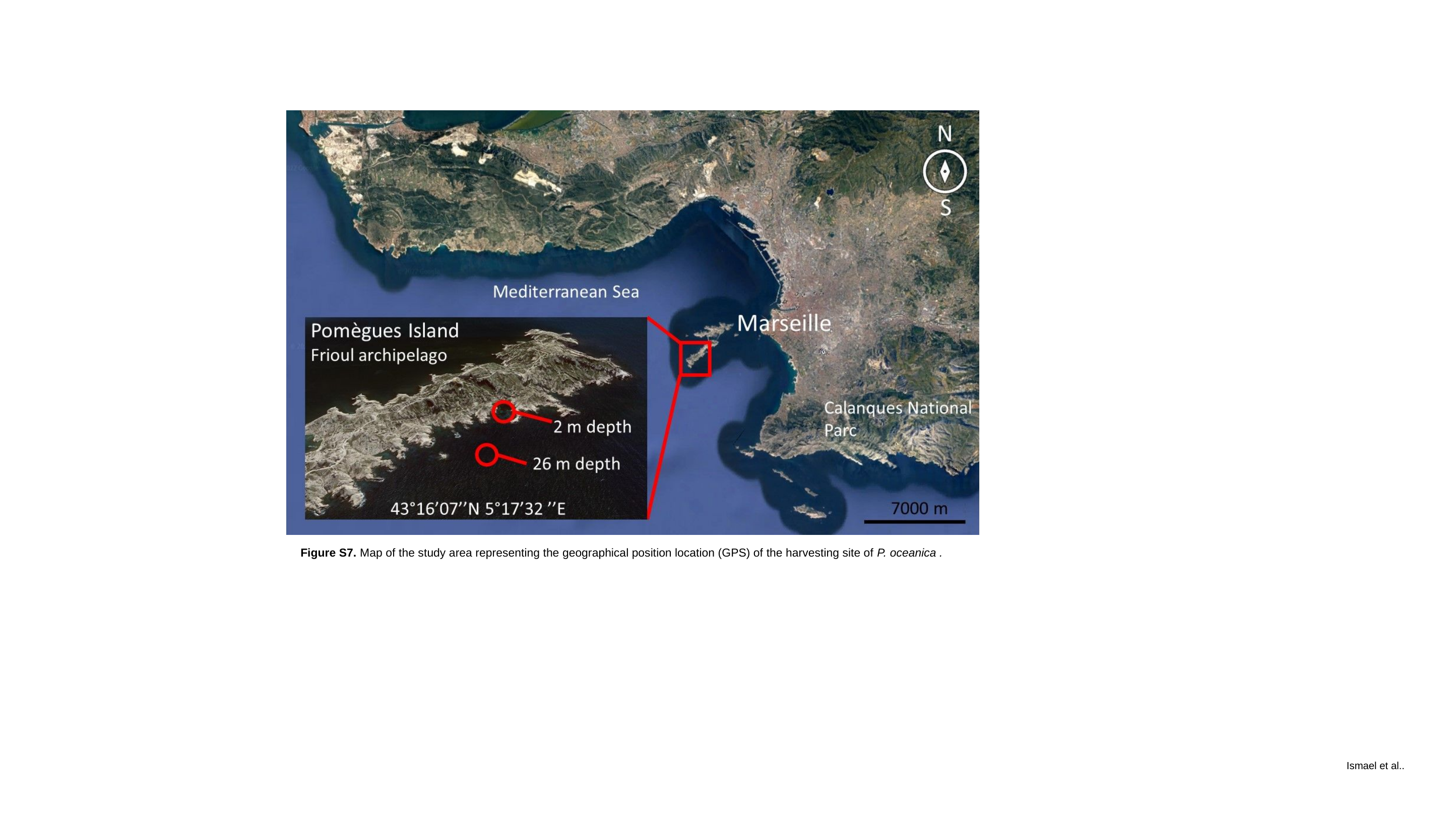

Figure S7. Map of the study area representing the geographical position location (GPS) of the harvesting site of P. oceanica .
Ismael et al..
